# Musical Sonification of Arm Movements in Stroke Rehabilitation Yields Limited Benefits

**DOI:** 10.3389/fnins.2019.01378

**Published:** 2019-12-20

**Authors:** Nikou Nikmaram, Daniel S. Scholz, Michael Großbach, Simone B. Schmidt, Jakob Spogis, Paolo Belardinelli, Florian Müller-Dahlhaus, Jörg Remy, Ulf Ziemann, Jens D. Rollnik, Eckart Altenmüller

**Affiliations:** ^1^Institute of Music Physiology and Musicians’ Medicine, Hanover University of Music, Drama and Media, Hanover, Germany; ^2^Institute for Neurorehabilitational Research (InFo), BDH-Clinic Hessisch Oldendorf, Associated Institute of Hannover Medical School (MHH), Hessisch Oldendorf, Germany; ^3^Department of Neurology & Stroke, Hertie-Institute for Clinical Brain Research, Eberhard Karls University of Tübingen, Tübingen, Germany; ^4^Department of Psychiatry and Psychotherapy, University Medical Center Mainz, Mainz, Germany; ^5^SRH Hochschule der Populären Künste (HDPK), Berlin, Germany

**Keywords:** sonification, stroke, neurorehabilitation, neuroplasticity, music-supported therapy, neurologic music therapy, auditory-motor coupling

## Abstract

Neurologic music therapy in rehabilitation of stroke patients has been shown to be a promising supplement to the often strenuous conventional rehabilitation strategies. The aim of this study was threefold: (i) replicate results from a previous study with a sample from one clinic (henceforth called Site 1; *N* = 12) using an already established recording system, and (ii) conceptually replicate previous findings with a less costly hand-tracking system in Site 2 (*N* = 30), and (iii) compare both sub-studies’ outcomes to estimate the efficiency of neurologic music therapy. Stroke patients in both sites were randomly assigned to treatment or control groups and received daily training of guided sequential upper limb movements additional to their standard stroke rehabilitation protocol. Treatment groups received sonification (i.e., changes in musical pitch) of their movements when they moved their affected hand up and down to reproduce a sequence of the first six notes of a C major scale. Controls received the same movement protocol, however, without auditory feedback. Sensors at the upper arm and the forearm (Xsens) or an optic sensor device (Leapmotion) allowed to measure kinematics of movements and movement smoothness. Behavioral measures pre and post intervention included the Fugl-Meyer assessment (FMA) and the Stroke Impact Scale (SIS) and movement data. Bayesian regression did not show evidence supporting an additional effect of sonification on clinical mobility assessments. However, combined movement data from both sites showed slight improvements in movement smoothness for the treatment group, and an advantage for one of the two motion capturing systems. Exploratory analyses of EEG-EMG phase coherence during movement of the paretic arm in a subset of patients suggested increases in cortico-muscular phase coherence specifically in the ipsilesional hemisphere after sonification therapy, but not after standard rehabilitation therapy. Our findings show that musical sonification is a viable treatment supplement to current neurorehabilitation methods, with limited clinical benefits. However, given patients’ enthusiasm during training and the low hardware price of one of the systems it may be considered as an add-on home-based neurorehabilitation therapy.

## Introduction

Stroke survivors frequently suffer from severe disabilities. Stroke may lead to impairments in motor and sensory systems, emotion regulation, language perception, and cognitive functions (Morris and Taub, 2008). Impaired arm function caused by gross-motor disability is also a common consequence of stroke immensely affecting quality of life in a considerable number of patients. In this case, regaining control over body movements is one of the crucial components in post-stroke recovery. There is an urgent need for effective motor rehabilitation approaches to improve quality of life in stroke survivors. Different therapeutic approaches such as Constraint Induced Movement Therapy (CIMT), mental practice, robot-aided therapy, electromyographic biofeedback, and repetitive task training have been applied to improve arm function after stroke (Langhorne et al., 2009). Of note, in a recent review it has been suggested that neurologic music therapy might be more effective than conventional physiotherapy (for a recent review see Sihvonen et al., 2017).

Motivational factors seem to play an important role for the beneficial effects of neurologic music therapy. From the patients’ informal descriptions of their experience with music-supported training, it appears that this is frequently highly enjoyable and a highlight of their rehabilitation process, regardless of the form of auditory stimulation, be it piano tones, or sonification of movement with other timbres [for a review see Altenmüller and Stewart (2018)]. However, effects of music supported therapy in stroke rehabilitation are not always consistent. In a recent review, seven controlled studies that evaluated the efficacy of music as an add-on therapy in stroke rehabilitation were identified (Sihvonen et al., 2017). In these studies, training of finger dexterity of the paretic hand was done using either a piano-keyboard, or, for wrist movements, drum-pads tuned to a C major scale. Superiority of the music group over fine motor training without music and over conventional physiotherapy was evident in one study after intervention comprising five 30-min sessions per week for 3 weeks (Schneider et al., 2010). The beneficial effect seen in the music group could be specifically attributed to the musical component of the training rather than the motor training *per se*, since patients practicing with mute instruments remained inferior to the music group. Here, the Fugl-Meyer Assessment (FMA) was applied before and after 20 sessions of either music supported therapy on a keyboard or equivalent therapy without sound. FMA scores of the motor functions of the upper limb improved by 16 in the music group and by 5 in the control group, both improvements being statistically significant although to a lesser degree in the control group (*p* = 0.02 vs. *p* = 0.04; Tong et al. (2015)).

With regard to the neurophysiological mechanisms of neurological music therapy, it was demonstrated that patients undergoing music supported therapy not only regained their motor abilities at a faster rate but also improved in timing, precision and smoothness of fine motor skills as well as showing increases in neuronal connectivity between sensorimotor and auditory cortices as assessed by means of EEG-EEG-coherence (Altenmüller et al., 2009; Schneider et al., 2010).

These findings are corroborated by a case study of a patient who underwent music supported training 20 months after suffering a stroke. Along with the clinical improvement, functional magnetic resonance imaging (fMRI) demonstrated activation of motor and premotor areas, when listening to simple piano tunes, thus providing additional evidence for the establishment of an auditory-sensorimotor co-representation due to the training procedure (Rojo et al., 2011). Likewise, in a larger group of 20 chronic stroke patients, increases in motor cortex excitability following 4 weeks of music-supported therapy were demonstrated using transcranial magnetic stimulation (TMS), which were accompanied by marked improvements of fine motor skills (Amengual et al., 2013).

In addition to functional reorganization of the auditory-sensorimotor network, recent findings have reported changes in cognition and emotion after music-supported therapy in chronic stroke patients. Fujioka et al. (2018) demonstrated in a 10-week-long randomized controlled trial (RCT), including 14 patients with music supported therapy and 14 patients receiving conventional physiotherapy, that both groups only showed minor improvements. However, the music group performed significantly better in the trail making test, indicating an improvement in cognitive flexibility, and furthermore showed enhanced social and communal participation in the Stroke Impairment Scale and in PANAS (Positive and Negative Affect Schedule, Watson et al., 1988), lending support to the prosocial and motivational effects of music. In another RCT with an intervention of only 4 weeks, Grau-Sánchez et al. (2018) demonstrated no superiority in fine motor skills in the music group as compared to a control group, but instead an increase in general quality of life as assessed by the Profile of Mood states and the stroke specific quality of live questionnaire. Despite growing evidence, the neurophysiological mechanisms of neurological music therapy remain poorly understood.

Most of the existing studies on music-supported therapy have focused on rehabilitation of fine motor functions of the hand. Much less evidence exists on post-stroke rehabilitation of gross motor functions of the upper limbs. In a previous study we thus developed a movement sonification therapy in order to train upper arm and shoulder functions (Scholz et al., 2015). Gross movements of the arm were transformed into discrete sounds, providing a continuous feedback in a melodic way, tuned to a major scale (i.e., patients could use movements of their paretic arms as a musical instrument). In this way, sound perception substituted for defective proprioception. In a first pilot study in subacute stroke patients we were able to demonstrate that musical sonification therapy reduced joint pain in the Fugl-Meyer pain subscale (difference between groups: −10; *d* = 1.96) and improved smoothness of movements (*d* = 1.16) in comparison to movement therapy without sound (Scholz et al., 2016). Here, we extend these findings by comparing the effects of the established musical sonification setup (Scholz et al., 2016) with a newly developed, less expensive sonification device in a group of subacute stroke patients with upper limb motor impairments. The only apparent differences between both data acquisition methods were the improved sound quality and the loss of need to strap sensors to patient limbs. In order to further elucidate the neurophysiological underpinnings of musical sonification therapy we simultaneously recorded EEG and EMG data from a subset of patients to analyze cortico-muscular phase coherence during upper limb movements (Chen et al., 2018; Pan et al., 2018). According to previous studies (Pan et al., 2018) we hypothesized that cortico-muscular phase coherence increases in the ipsilesional hemisphere after musical sonification therapy.

## Materials and Methods

### Patients

Patient inclusion criteria were acute or subacute unilateral stroke on one hemisphere, and decided on by the admitting physician based on the clinical picture of the patients. No other screening tools or cut-offs were used. Exclusion criteria were reports of aphasia, additional neurological, psychiatric or cognitive deficits. Moreover, patients needed to be able to perform gross motor arm movements without the assistance of their unaffected side’s limb.

For Site 1, one patient was enrolled at ZAR Tübingen, Germany (center for outpatient rehabilitation), and 11 patients were enrolled at M&I Clinics Hohenurach, Bad Urach, Germany. At BDH Clinic Hessisch-Oldendorf, Germany, henceforth called Site 2, 30 patients were enrolled. Two patients at Site 2 were excluded due to data loss or loss to follow-up, respectively.

**FIGURE 1:**
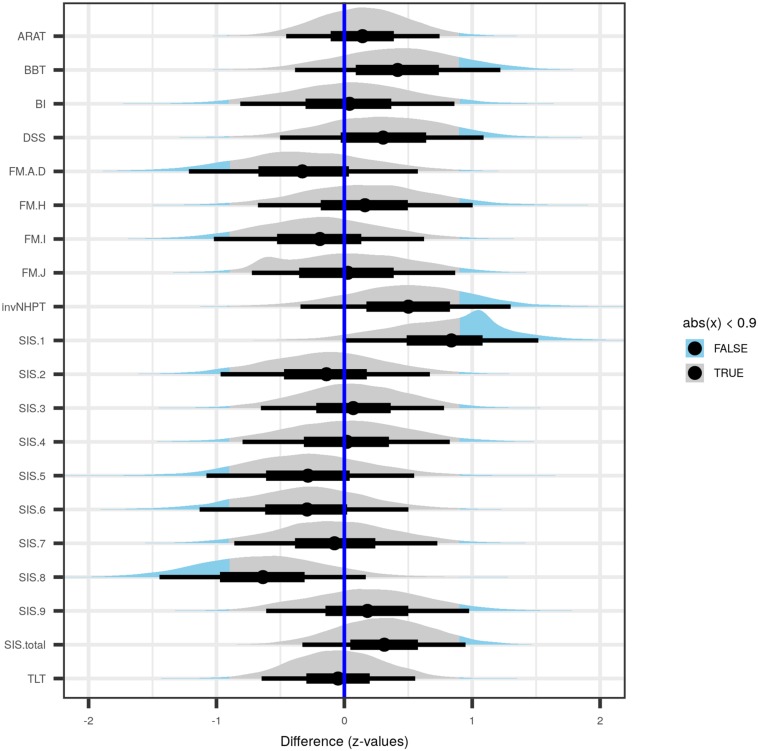
Posterior distributions of differences between treatment and control groups of the tested variables, each along with median point estimate (dot), 50 percent uncertainty interval (thick black horizontal bar), and 90 percent uncertainty interval (thin black horizontal bar). The part of a given distribution further than 0.9 standard deviations away from zero is shown in light blue instead of in gray. Point estimates >0 represent higher initial scores in the treatment group, while values <0 imply larger initial scores in the control group. ARAT, Action Research Arm Test; BBT, Box and Block Test; BI, Barthel Index prior to begin of rehabilitation; DSS, number of days elapsed between ocurrence of stroke and commencement of rehabilitation; FM.A-D, Fugl-Meyer test subscales A–D, covering reflexes, volitional movements, wrist and hand function and the coordination of the upper extremity; FM.H, tactile sensation in the affected and non-affected extremity; FM.I, passive joint motion; FM.J, passive movement joint pain; invNHPT, (inverted) Nine-Hole Peg Test; SIS.1, physical problems as a result of the stroke; SIS.2, memory and thinking abilities; SIS.3, mood and emotions; SIS.4, communicational skills in speaking, reading and writing; SIS.5, impairment of daily activities; SIS.6, mobility; SIS.7, remaining function of the affected hand; SIS.8, impairment of social activities; SIS.9, self-rating of how far stroke recovery has progressed; TLT, Thumb Localizing Test; SIS.total, total sum score over Stroke Impact Scale subscales. See “Materials and Methods” section for details.

Patients were alternatingly assigned to either control or treatment group in the order of enrollment at Site 1, and pseudo-randomly assigned at Site 2 to the experimental or to the control group by the supervisor of the study who was not the experimenter. Both treatment groups received conventional physiotherapy plus a musical sonification training. The control groups also received conventional physiotherapy and an additional sham sonification movement training with exactly the same movements required as in the sonification group, but with no sound being played back. All patients were German native speakers. See Table 1 and Figure 1 for patient characteristics and group differences.

**TABLE 1:** Patient characteristics.

	**Site 1**		**Site 2**	
		
	**Treatment**	**Control**	**Treatment**	**Control**
*N*	7	5	14	14
Male	6	3	10	11
Age, M ± SD; range, years	65.30 ± 12.70; 50–84	66.40 ± 6.90; 59–76	68.71 ± 11.76; 48–92	70.21 ± 14.29; 42–88
Right arm affected	3	1	5	6
Right-handed	7	5	14	14
Days after stroke median (range)	27 (16–40)	21 (18–27)	36.5 (12–144)	26 (5–510)
Training days, median (range)	15 (11–15)	15 (13–15)	22 (7–40)	16.5 (9–46)
Barthel index, M ± SD	45.70 ± 23.20	42.00 ± 20.20	39.64 ± 17.27	36.43 ± 17.87
**Fugl-Meyer Assessment: median (range)**				
FM.A-D,	39 (13–50)	44 (25–48)	52.5 (20–65)	55.5 (14–65)
FM.H,	12 (10–12)	12 (8–12)	12 (2–12)	12 (8–12)
FM.I,	24 (23–24)	24 (24–24)	24 (12–24)	24 (18–24)
FM.J,	23 (22–24)	24 (22–24)	24 (12–24)	24 (18–24)
**Lesion type:**				
Ischemic/hemorrhagic	7/0	5/0	12/2	9/5
**Lesion site:**				
Left cortical	1			
– Frontal				
– Fronto-temporal with participation gyrus pre- and post-centralis				1
– Occipital				1
– Parietal			1	1
– Temporal				2
– A. Cerebri media flow area		1	2	1
**Left subcortical**				
– Capsula interna			1	
– Basal ganglia			1	
Left pons	2			
Right cortical	1			
– Frontal			2	2
– Fronto-parietal				4
– Occipital			1	1
– Parietal			1	
– Parietooccipital			3	
– Temporal				
– A. Cerebri media flow area	1			1
**Right subcortical**				
– Capsula interna	1			
– Basal ganglia		1	1	
Right pons	1	3	1	

*Total number *N* of patients per group are given per site; age at the begin of the study, the number of patients affected by stroke in the right arm, and the number of right-handed patients are provided; the number of training sessions patients were subjected to and the patients’ mean Barthel index prior to study commencement are also supplied. Scoring on Fugl-Meyer subscales as well as lesion types and lesion sites are specified.*

This study was carried out in accordance with the recommendations of the Ethics Review Board of the Hannover Medical School and the Ethics Committee of the Medical Faculty of Eberhard Karls University of Tübingen. The protocol was approved by the Ethics Review Board of the Hannover Medical School (Approval No. 1767-2013) and the Ethics Committee of the Medical Faculty of Eberhard Karls University of Tübingen (Protocol No. 597/2013BO2). All subjects gave written informed consent in accordance with the Declaration of Helsinki.

### Experimental Setup

**FIGURE 2:**
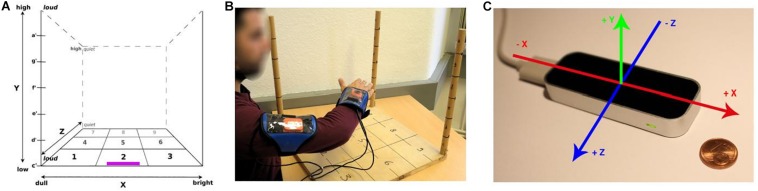
Experimental setup. **(A)** three-dimensional space (the Leapmotion controller at Site 2 was placed on the board at the position marked in purple), with axis labels describing qualitative sound changes when the hand was moved relative to the frame (and hence, the body). **(B)** Xsens sensors as used at Site 1, attached to wrist and upper arm of patient. **(C)** Leapmotion controller as used at Site 2, with the space axes superimposed. Panel **(A)** taken from Scholz et al. (2016).

Training took place as regular one-on-one sessions (see *training days* in Table 1), in which patients sat in front of a table with a wooden frame on top. The frame consisted of a 51 × 51 cm board at the bottom that was subdivided into nine equally spaced numbered fields (Figure 2A) to simplify instructions where in the horizontal plane a task had to be carried out. Vertical bars (length: 51 cm) were attached in three corners of the board, all subdivided by clearly visible markings into six equally spaced intervals. Each interval was labeled with a musical note-pitch name of the C major scale from c’ (at the bottom) to a’ (top). Tasks increased in complexity throughout each session and consisted of up-and-down movements of the hand at one position in the *x*-*z* plane. Up-and-down movement instructions for each task were shown separately as a sequence of musical note pitches on a sheet behind the frame. The tasks consisted of four upward and downward legato C major scales, restricted to the first six notes (i.e., c′-d′-e′-f′-g′-a′ and g′-f′-e′-d′-c′) at each of the positions 1, 2, 7, and 9 (Figure 2A) as well as musical intervals from c′ to d′, from c′ to e′, from c′ to f′, from c′ to g′, and from c′ to a′. This exercise was also repeated four times at positions 1, 2, 3, 7, and 9. The final goal of the training was to teach patients to play several simple nursery rhymes or other familiar tunes only by moving their affected arm in the three-dimensional sonification space. Patients always moved their impaired arms by themselves without the aid of neither their unimpaired arm nor the experimenter.

Patients at Site 1 wore Xsens inertial sensors (model X-MB-XB3; Figure 2B)^1^ at the wrist and upper arm that transmitted acceleration, rotation, and gravity data via Bluetooth® to a computer with custom-made software that inferred the current coordinates of the hand relative to the dimensions of the wooden frame and mapped the thus determined position to a predefined sound.

At Site 2, a Leapmotion controller (Figure 2C)^2^ was located at the edge of the front of the board. The controller consists of three infrared light emitters and two infrared cameras and tracks hand movements in three dimensions. The controller transferred the coordinates of the patient’s palm centroid within the predefined space to a custom-made computer program on a computer. There the coordinates were mapped to the corresponding sound parameters which were subsequently played back to the patient in real-time.

Note pitches ranged from c′ = 226.6 Hz at the bottom to a′ = 440 Hz at the top. On the *x* axis, sound varied in brightness via a variation in sound synthesis (Site 1; Synthesis ToolKit, Cook and Scavone, 1999) or of the sound samples used (Site 2) with three different timbres (from dull = clarinet sound on the left side of the patient, to saxophone in the middle, and a bowed instrument = bright at the right). Loudness of sounds was mapped along the *z* axis, so that a proximal hand position resulted in a louder sound than a more distal one. Regular training sessions lasted approximately 30 min.

### Evaluation of Motor Functions and Stroke Impact

Evaluation of the patient rehabilitation process was conducted by administering several clinical motor function tests and a psychological questionnaire pre and post training.

The clinical motor function tests consisted of six major sections:

(a) The upper extremity part of the FMA, still considered the gold standard for the evaluation of motor recovery after stroke (Crow and Harmeling-van der Wel, 2008; Woodbury et al., 2008). The FMA includes four main subsections
  • FM.A-D: reflexes, volitional movements, wrist and hand function and the coordination of the upper extremity
  • FM.H: tactile sensation in the affected and non-affected extremity
  • In FM.I: passive joint motion
  • FM.J: passive movement joint pain
(b) The Box and Block Test (BBT) assesses unilateral gross manual dexterity (Mathiowetz et al., 1985; Canny et al., 2009; Chen et al., 2009)
(c) The Nine-Hole Peg Test measures finger dexterity (Grice et al., 2003). For modeling purposes and to simplify presentation of data, the obtained scores were inverted (invNHPT)
(d) The Stroke Impact Scale (SIS; Duncan et al., 2003; Lin et al., 2010) evaluates health status following a stroke, including sub-scales for emotional well-being, memory, thinking and social participation. The consecutively numbered subscales are
  (1) physical problems as a result of the stroke
  (2) memory and thinking abilities
  (3) mood and emotions
  (4) communicational skills in speaking, reading and writing
  (5) impairment of daily activities
  (6) mobility
  (7) remaining function of the affected hand
  (8) impairment of social activities
  (9) self-rating of how far stroke recovery has progressed
(e) Thumb Localizing Test (TLT; Hirayama et al., 1999)
(f) The Action Research Arm Test (ARAT; Lyle, 1981)

Additionally, the Barthel Index (BI; Mahoney and Barthel, 1965) prior to intervention, and the number of days between the occurence of the stroke and beginning of the intervention (Days Since Stroke–DSS) were collected. Administration of the motor assessment test battery and the questionnaire pre and post intervention took approximately 1 h to complete.

### EMG and EEG Recordings

At Site 1, electrophysiological data were acquired from two subjects who underwent music therapy (both left hemispheric stroke, trained on right arm) and two subjects who underwent control therapy (one left hemispheric stroke, trained on right arm; one right hemispheric stroke, trained on left arm), before and after therapy. Subjects were instructed to conduct one hundred self-paced elevations of their paretic and non-paretic arm, respectively, in separate blocks of trials, and at a frequency of around one elevation per 5 s. Specifically, subjects were asked to elevate their arm from c’ to d’ in the *y*-axis at position 1 (right arm) and position 3 (left arm), respectively, in the three-dimensional training space (cf. Figures 1A,B), along with training of upward C major scale movements during therapy. EMG (from deltoid muscles) and 20-channel EEG were recorded using a Neurofax EEG-9200 system (Nihon Kohden, Japan). The position of the EEG electrodes followed the International 10–20 system (Seeck et al., 2017), and EEG data were referenced to A1 and A2 (linked earlobes). Biosignals were recorded at a sampling frequency of 200 Hz. Electrode impedances were regularly checked and kept below 10 kΩ throughout the experiment.

### Movement Smoothness

Movement trajectories from the patients’ first task (four C major scales at position 1) on each training day were manually identified and separated into upward and downward strokes for offline calculation of movement smoothness. Following Osu et al. (2011), in each of these strokes the three-dimensional curvature κ^2^ for each time point was determined:

$$\kappa^{2}=\frac{\left({\dot{x}}^{2}+{\dot{y}}^{2}+{\dot{z}}^{2}\right)\,\left({\ddot{x}}^{2}+{\ddot{y}}^{2}+{\ddot{z}}^{2}\right)-{\left(\dot{x}\,\ddot{x}+\dot{y}\,\ddot{y}+\dot{z}\,\ddot{z}\right)}^{2}}{\left({\dot{x}}^{2}+{\dot{y}}^{2}+{\dot{z}}^{2}\right)}$$

The median of the negative natural log from the κ^2^ vector of each stroke was taken as a measure of its movement smoothness.

### Data Analysis

EEG and EMG data which were analyzed using MATLAB (version R2017b, The MathWorks) with EEGLAB (version 13.5.4b; Delorme and Makeig, 2004) and Fieldtrip (Oostenveld et al., 2011) toolboxes. The programming language R (version 3.5.1; R Core Team, 2018) in conjunction with RStudio Server (version 1.2.1080; RStudio Team, 2018) was used for all other data preprocessing and analyses.

To account for small samples, unbalanced group sizes, and a decreasing number of data points over time (see “Results” section on data loss) we opted for Bayesian multilevel regression modeling to analyze the motor test outcomes and movement smoothness data. Modeling was carried out with the R package brms (Bürkner, 2018). In Bayesian regression, small samples can be bridled by using informative priors, while the growing uncertainty about the distribution of estimated parameters due to the diminishing number of data points over time, e.g., caused by data loss or dropout, is reflected by increasingly wider credible intervals, acknowledging the growing uncertainty. Multilevel modeling (MLM) also helps keeping the lid on small clusters by partial pooling, which basically leads to shrinkage of lower level estimates toward higher level estimates. If, for instance the highest level of a MLM is the grouping into treatment and controls, then group averages can be estimated based on the grand-average. A far-off group estimate is then shrunk toward the grand-average, and the more so, the fewer data points this extreme estimate contains (Gelman et al., 2012).

#### Motor Test Batteries and the Stroke Impact Scale

Simple Bayesian regressions were carried out for all motor test battery subscales prior to intervention, with *Treatment* (0|1) as predictor, to determine any differences between the two groups prior to intervention. Pre-intervention scores of outcome variables were *z*-transformed to increase computational stability, and priors were chosen to be informative with heavy tails to allow for extreme values (central Student’s *t* distribution: *df* = 3; *scale* = 1; left-bounded at zero for variance parameters).

Posterior distributions of pre-intervention differences between treatment and control groups in the motor test batteries, the SIS scores, and the number of days between stroke and begin of the intervention are shown in Figure 1. Almost all of the difference-distributions substantially overlap with zero, with the notable exceptions *SIS.1*, which was larger in the treatment group, while *SIS.8* was larger in the control group.

#### Movement Smoothness

Increasingly complex multilevel models were built using the joint data sets from both sites in order to examine and better understand the underlying data-generating processes. All models included *z*-transformed movement smoothness (zero mean and unit *SD*) as outcome. The most simple model used patient group as population-level predictor, while further models included an increasing number of explanatory variables, eventually modeling correlated varying coefficients (see Table 2). All priors were chosen to be informative; slopes were modeled to be student *t*-distributed with *df* = *3*, located at zero, and with scale set to 3, and left-bounded at zero for variance components; priors placed on varying parameter correlation matrices were LKJcorr, with η = 2. Prediction accuracy between models was compared using Pareto-smoothed importance sampling leave-one-out cross-validation (Vehtari et al., 2017), an approximation to leave-one-out cross-validation.

**TABLE 2:** Parameter estimates of models used to explain movement smoothness over time in treatment and control groups, using two different motion capturing systems.

**Number**	**Model**	**Term**	**estimate**	**std.error**	**conf.low**	**conf.high**
1	MedianLC ∼ Group.c + (1 | IDanon)	Intercept	–0.17	0.13	–0.39	0.05
		Group.c	0.49	0.26	0.04	0.91
2	MedianLC ∼ Group.c × Session.c + (1 | IDanon)	Intercept	–0.17	0.13	–0.39	0.05
		Group.c	0.49	0.26	0.05	0.90
		Group.c:Session.c	0.00	0.03	–0.04	0.05
		Session.c	0.01	0.01	–0.02	0.03
3	MedianLC ∼ Group.c × Session.c + (Session.c | IDanon)	Intercept	–0.15	0.14	–0.38	0.07
		Group.c	0.50	0.26	0.07	0.92
		Group.c:Session.c	0.01	0.03	–0.04	0.06
		Session.c	0.01	0.02	–0.02	0.04
4	MedianLC ∼ Group.c × Session.c + pre.z + (Session.c + pre.z | IDanon)	Intercept	–0.10	0.10	–0.27	0.05
		Group.c	0.23	0.19	–0.09	0.55
		Group.c:Session.c	0.00	0.03	–0.05	0.04
		Session.c	0.01	0.01	–0.01	0.04
		pre.z	0.56	0.12	0.36	0.77
5	MedianLC ∼ Group.c × Session.c + pre.z + MoCap.c + (Session.c + pre.z | IDanon)	Intercept	–0.10	0.09	–0.25	0.06
		Group.c	0.21	0.19	–0.09	0.52
		Group.c:Session.c	0.00	0.03	–0.05	0.04
		Session.c	0.00	0.02	–0.02	0.03
		pre.z	0.54	0.12	0.34	0.74
		MoCap.c	–0.34	0.20	–0.66	–0.03

*Models grow in complexity from top to bottom of the table. The outcome variable in every model is the median negative natural logarithm of movement curvature (term MedianLC in the Model column). To account for repeated measurements, each model incorporates an individual varying intercept for each patient (term | IDanon). The term Group.c represents the estimated average of the two groups. Model 2 also incorporated training sessions the patients partook (Session.c); model 3 added an interaction between Group.c and Session.c. Model 4 allowed the session slope of each individual to correlate with their intercept: (Session.c | IDanon). Models 5 and 6 also accounted for standardized pre-treatment movement smoothness (pre.z) and the type of motion capturing system (MoCap.c), respectively. The column std.error lists the standard error, while columns conf.low and conf.high contain the 90 percent credible interval bounds of the point estimate.*

#### EMG and EEG

EMG event markers for movement onset were set manually by visual inspection and using an individually adjusted threshold of 30–110 μV according to individual noise levels. Post therapy EMG data from one subject who underwent control therapy was too noisy to allow for reliable identification of movement onsets; this subject was excluded from further analysis. Data of the remaining three subjects (two left hemispheric stroke patients with music therapy, one right hemispheric stroke patient with control therapy) were analyzed using MATLAB (R2017b, MathWorks) and the Fieldtrip open-source toolbox (Oostenveld et al., 2011), with customized scripts. Trials were visually inspected and noisy trials were removed from further analysis. For the patients with music therapy, 96 epochs before (pre) and 98 epochs after (post) therapy, and 98 epochs for both pre and post therapy measurements, respectively, were considered; for the patient with control therapy, 85 trials for both pre and post therapy measurements were considered. Data were first detrended and band-pass filtered between 2 and 80 Hz. Subsequently, data were filtered with a 1–80 Hz 3rd order Butterworth zero phase band pass filter and a 49–51 Hz notch filter. As a measure of cortico-muscular phase coherence the Weighted Phase Lag Index (WPLI) was computed between EEG and EMG channels, following previous reports (Stam et al., 2007; Vinck et al., 2011). As phase coherences were found significant (Rosenberg et al., 1989) in channels of the sensorimotor area (i.e., electrodes C3 and C4, respectively) pre and post therapy with a 95% confidence probability only in the low beta (14–20 Hz) frequency band, further analyses were restricted to the low beta band.

A cluster-based permutation analysis (Maris et al., 2007) was performed to test for significant differences between beta band cortico-muscular phase coherence pre vs. post therapy. Cluster statistics were evaluated at the single subject level, considering each trial as a unity of observation. The minimum number of neighboring channels to form a cluster was 2. A positive cluster was defined as WPLI Post > WPLI Pre, a negative cluster as WPLI Post < WPLI Pre. The significance level was set at *p* < 0.05.

## Results

### Motor Test Batteries and the Stroke Impact Scale

**FIGURE 3:**
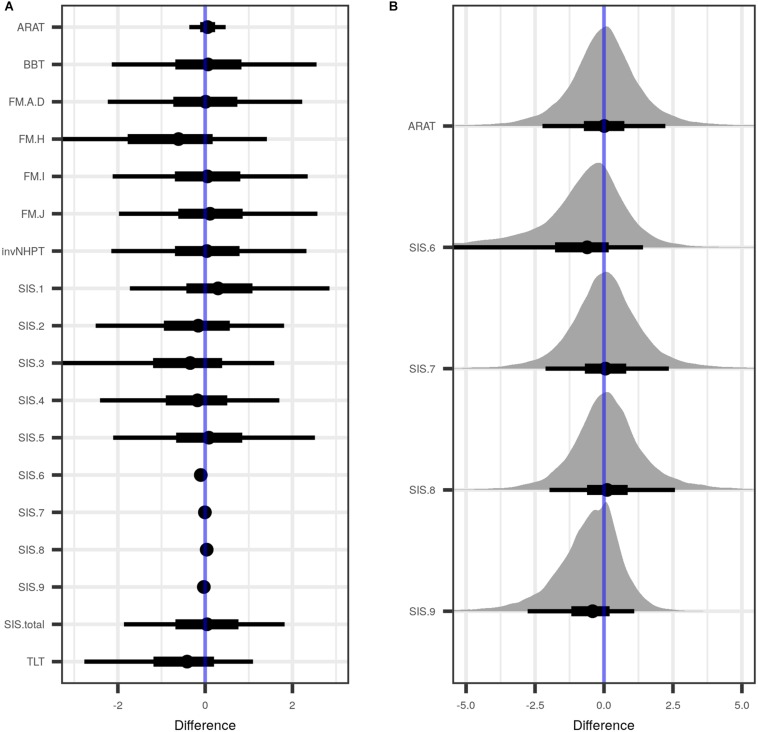
Estimated differences between treatment and control group, when data from both sites were combined and pre-treatment values were taken into account. **(A)** Shown are the posterior distributions of differences between treatment and control groups of the tested variables, with median point estimate (dot), 50 percent uncertainty interval (thick black horizontal bar), and 90 percent uncertainty interval (thin black horizontal bar). **(B)** A close-up of those variables in A with a point estimate close to zero. ARAT, Action Research Arm Test; BBT, Box and Block Test; FM.A-D, Fugl-Meyer test subscales A–D, covering reflexes, volitional movements, wrist and hand function and the coordination of the upper extremity; FM.H, tactile sensation in the affected and non-affected extremity; FM.I, passive joint motion; FM.J, passive movement joint pain; invNHPT, (inverted) Nine-Hole Peg Test; SIS.1 (Stroke Impact Scale, subscale 1), physical problems as a result of the stroke; SIS.2, memory and thinking abilities; SIS.3, mood and emotions; SIS.4, communicational skills in speaking, reading and writing; SIS.5, impairment of daily activities; SIS.6, mobility; SIS.7, remaining function of the affected hand; SIS.8, impairment of social activities; SIS.9, self-rating of how far stroke recovery has progressed; TLT, Thumb Localizing Test; SIS.total, total sum score over SIS. See “Materials and Methods” section for details.

Post-treatment differences in motor test batteries and the SIS are depicted in Figure 3, while Supplementary Table S1 lists posterior point estimates along with credible intervals, separately for both sites. All difference point estimates lay close to zero, and most had very wide credible intervals, the latter pointing to large heterogeneity of the data which impeded more precise estimation given the sample.

### Movement Smoothness

Smoothness assessing models did not converge for data from Site 1 alone, most likely due to a combination of pronounced heterogeneity and small sample size. A considerable amount of smoothness data at site 2 was lost due to a combination of human and technical error. While patients adhered to the regular training schedule with a median of 22 (treatment group) and 16.5 (control group) training sessions (see also *training days* in Table 1), the data available for analysis only had a median (range) of 2.5 (1,7) sessions. We therefor decided to pool the movement data from both sites.

**FIGURE 4:**
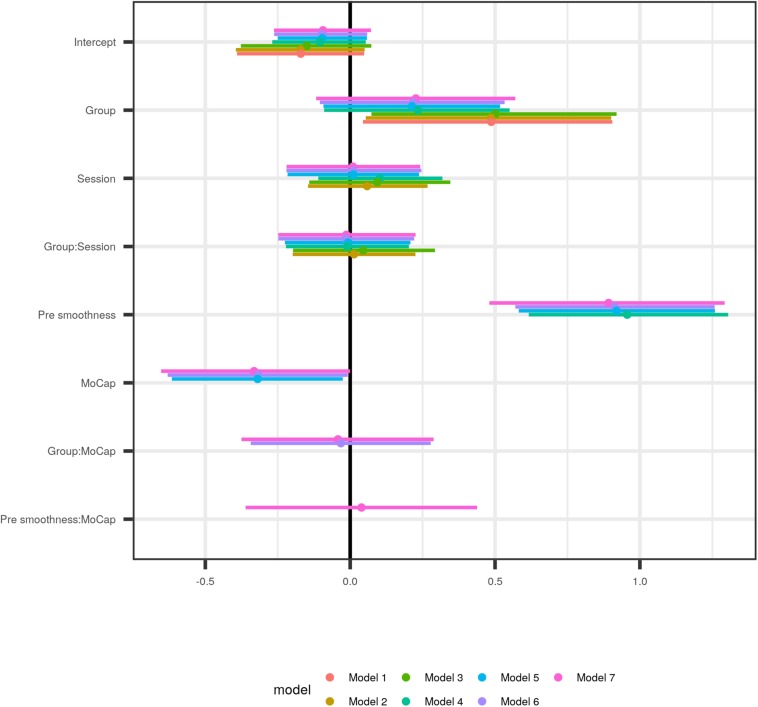
Comparison of population parameters as estimated by models of increasing complexity, starting with the most simple one (#1), which modeled the outcome as the result of Group, and hence only has two parameters (Intercept and Group; see Table 2 for a numerical representation of the data). Model 2 additionally employed the time factor (Session) as explanatory variable, and its interaction with Group, and thus has two further parameter estimates, and so forth. The first three models do not change considerably, only the addition of pre-intervention movement smoothness pre smoothness as covariate in model 4 moves the Group estimate closer to zero. In model 5, with the motion capturing system added as covariate (MoCap), the Group estimate does not change much although the MoCap effect is noticeable.

In the combined smoothness data set, the most simple model estimated a substantial average smoothness increase for both groups [approx. 0.5 (CI: 0, 0.9) standard deviations], but with a wide credible interval (model #1 in Figure 4 and Table 2). The subsequent addition of *Session*, and the interaction *Group:Session* (model #2) as regression input, as well as modeling the correlation between an individual’s intercepts and their session slopes (model #3) did not considerably change the estimate of the average group effect. Both the effect of *Session*, and the interaction *Group:Session* were estimated to be close to zero, with 90%-credible intervals substantially overlapping with zero, indicative of non-relevant smoothness changes across sessions. However, the addition of pre-treatment smoothness as a covariate (model #4) led to a decent decrease in the estimated average group effect. Adding the type of motion capture device employed at a given site (*MoCap*, #5) did not further change the group estimate, nor did it lead to an increase in predictive accuracy (Supplementary Table S2). Regardless, the effect of *MoCap* was not negligible. This last result led us to *post hoc* model the interaction *Group:MoCap* (#6), which was estimated to be very close to zero. The unexpected effect of *MoCap* led to further exploration of the interaction *Pre smoothness:MoCap* which was estimated to be close to zero (#7).

**FIGURE 5:**
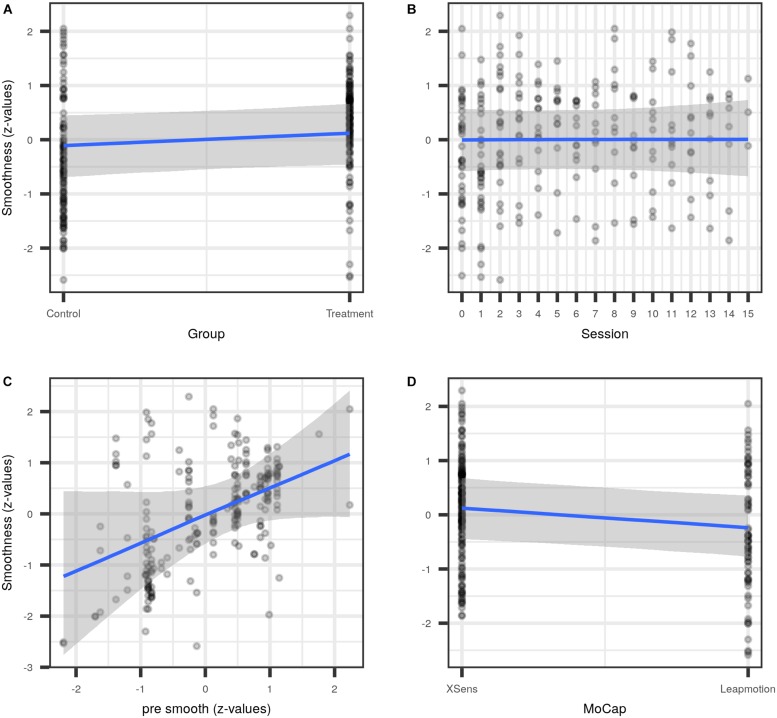
Conditional plots for model #5. Population effects for the covariates Group **(A)**, medianized session **(B)**, standardized pre-intervention smoothness pre smooth **(C)**, and the motion capturing system MoCap **(D)**.

While in non-Bayesian regression adjusted R^2^ serves as a measure against overfitting, prior probabilities in Bayesian models and shrinkage in MLM jointly serve the same purpose, together with estimated leave one out cross-validation of the posterior log-likelihood (PSIS-LOO-CV; Vehtari et al., 2017). The latter is also used to find the best fitting model by identifying the expected log predictive density (elpd) differences between models. See Supplementary Table S2. Figure 5 shows conditional plots of the population-level effects for model #5, placing the parameter estimates from Figure 4 into context of the data.

The best-fitting model (#5) estimated an average movement smoothness increase for *Group* of 0.21 [−0.09, 0.52]; see Table 2.

### Cortico-Muscular Phase Coherence

**FIGURE 6:**
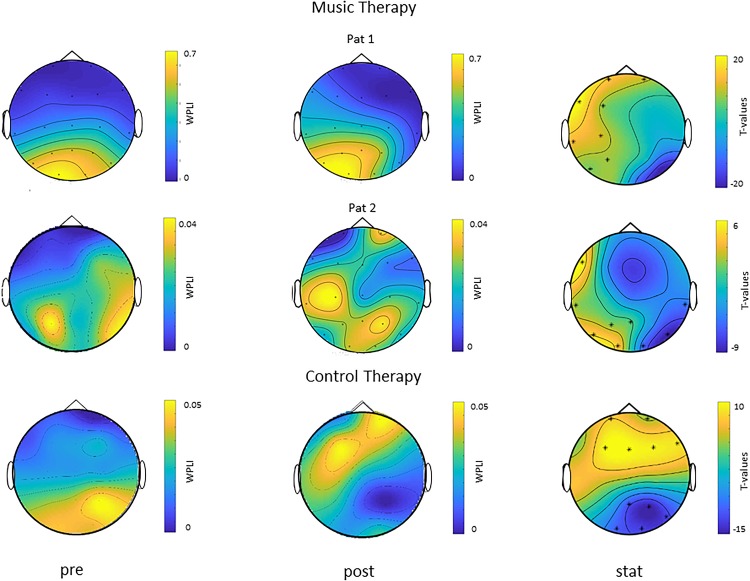
Modulation of cortico-muscular phase coherence during movement of the paretic arm (single subject data). Topoplots of WPLI as a measure of cortico-muscular phase coherence before (pre) and after (post) therapy in two patients with music therapy (Pat 1 and Pat 2, both left hemispheric stroke, right arm trained, upper two rows) and one patient with control therapy (right hemispheric stroke, left arm trained, lower row) are shown in the left and middle column, respectively. T-statistic maps (stat) of cluster-based analyses of differences in WPLI post vs. pre therapy are shown in the right column. Channels belonging to significant clusters are marked with asterisks. Note left hemispheric (i.e., ipsilesional) cortico-muscular phase coherence increases during movements of the paretic right arm in the two patients with music therapy, while the patient with control therapy showed a rather bilateral topography of cortico-muscular phase coherence modulations during movements of the paretic left arm.

Exploratory analyses of beta-band EEG-EMG phase coherence as measured with WPLI during movement of the paretic arm showed a positive EEG cluster in the left (lesioned) hemisphere (channels: Fp1, Fp2, F3, C3, P3, F7, T3, T5) for patient 1 with music therapy, and a positive cluster in the left (lesioned) hemisphere (channels: F7, T3, T5, P3, O1, Pz) and a negative right posterior cluster (channels: O2, T6, T4) for patient 2 with music therapy (Figure 6). These findings indicate increased cortico-muscular phase coherence post vs. pre musical sonification therapy in the ipsilesional hemisphere during movement of the paretic arm. In contrast, for the patient with control therapy, who had suffered a right hemispheric stroke, a positive frontal cluster (channels: Fp1, Fp2, F3, Fz, F4, F8) and a negative posterior cluster (channels: Pz, P4, T6, O1, O2) with bilateral topography were found in post vs. pre therapy comparisons. Of note, for movements with the non-paretic arm no significant cluster was found in either patient (data not shown), indicating similar cortico-muscular phase coherences (i.e., WPLI values) at the two measurement time points during movement of the non-trained arm in all patients.

## Discussion

Summarizing the effects of rehabilitation of the upper limb after stroke, it is unclear whether musical sonification training is efficient. Bayesian regression of several motor test batteries and the Stroke Impairment Scale did not provide evidence supporting an additional effect of the treatment. However, MLM revealed that movement smoothness of the treatment group was greater, albeit with the credible interval overlapping zero (Figures 4, 5). This suggests a small effect, if any, of musical sonification training on movement smoothness. Adding pre-treatment smoothness as covariate to the model (model 4) decreased the *Group* effect estimate substantially. This suggests that the heterogeneity of the pre-treatment movement smoothness in the sample considerably influenced the accuracy with which the improvement could be estimated. When the type of motion capturing system was added to the model as predictor (model 5), it captured a substantial amount of variation. This last point may be explained in several, not mutually exclusive ways. It is possible that the samples at both sites either differed in more respects than had been anticipated, and this difference was not apparent in the pre-treatment screening (Figure 2). Or, differential handling of patients at the two sites may have led to differing success of the supplementary rehabilitation. A third possible explanation, and one corroborated by our data, would be the differing temporo-spatial resolution of the two motion capturing systems (Figure 5D), resulting in the Leapmotion sensor finding movements of comparable groups “rougher” then would the Xsens system.

Exploratory analyses of EEG-EMG coherence during movement of the paretic arm in a subset of our patients suggested increased beta-band cortico-muscular phase coherence specifically in the lesioned hemisphere after musical sonification therapy, but not after motor training without sonification (cf. Figure 6). Of note, cortico-muscular phase coherence during movement of the non-paretic arm did not change after either training. These findings are in line with previous results showing an increase in beta-band cortico-muscular phase coherence in the lesioned hemisphere after 4 weeks of electrical stimulation of the median nerve combined with hand function training, but not after hand function training alone (Pan et al., 2018). Whether increases in beta-band cortico-muscular phase coherence indeed underlie clinical improvements of motor function of the upper limb after musical sonification therapy, and how these changes link to functional reorganization of the auditory-sensorimotor (Altenmüller et al., 2009; Schneider et al., 2010; Fujioka et al., 2012) or other (e.g., fronto-parietal) cerebral networks needs to be investigated in future studies.

Several other mechanisms have been implicated in the effects of music-supported therapy of motor function post-stroke. From the patients’ informal descriptions of their experience with music-supported training, it appears that this was highly enjoyable and a highlight of their rehabilitation process, regardless of the form of auditory stimulation. Thus, as already explored in earlier articles, motivational and emotional factors might have contributed to the improvement of the training program (as reported in Särkämö et al., 2008). In addition, the role of the auditory feedback in music-supported therapy needs further investigation. Up to now it has not been clarified whether auditory feedback *per se* (e.g., simple beep tones) can have a similar effect on fine motor post-stroke rehabilitation, or whether explicit musical parameters such as a sophisticated pitch and time structure are prerequisites for the success of the training. This has to be addressed in a study comparing the effects of musical feedback compared to simple acoustic feedback. With respect to the latter, according to a study by Thaut et al. (2002), simple rhythmic cueing with a metronome significantly improves the spatio-temporal precision of reaching movements in stroke patients.

Finally, it is not clear whether timing regularity and predictability is crucial for the beneficial effect of music supported therapy. Although it has been argued that the effectiveness of this therapy relies on the fact that the patient’s brain receives a time-locked auditory feedback with each movement, new results challenge this viewpoint. In a recent study, 15 patients in early stroke rehabilitation with no previous musical background were studied (van Vugt et al., 2016). They learned to play simple finger exercises and familiar children’s songs on the piano. Participants were assigned to one of two groups: in the normal group, the keyboard emitted a tone immediately at keystroke, in the delay group, the tone was randomly delayed. To assess recovery, standard clinical tests such as the nine-hole-pegboard test and index finger tapping speed and regularity were used. Surprisingly, patients in the delay group improved in the nine-hole-pegboard test, whereas patients in the normal group did not. The normal group showed reduced depression whereas the delay group did not. Thus, music supported therapy even with a randomly delayed keyboard can improve motor recovery after stroke, possibly because patients in the delayed feedback group implicitly learn to be independent of the auditory feedback and therefore outperform those in the normal condition when auditory feedback is not available.

In summary, musical sonification therapy for rehabilitation of motor impairments of the upper limbs is a viable treatment option, yet with limited clinical effects in subacute stroke patients. Given the patients’ enthusiasm during training and the low hardware price for one of the sonification devices it may be considered as an add-on, home-based neurorehabilitation therapy. Future research should address the long-term sustainability of improvements and strive to optimize length and number of training sessions, according to patients needs and preference. Most probably, a client-tailored treatment algorithm considering severity of impairment, psychological status and motivational drive would be most efficient.

## Data Availability Statement

All datasets generated for this study are included in the article/Supplementary Material.

## Ethics Statement

The study was approved by the Ethics Review Board of the Hannover Medical School (Approval No. 1767-2013) and the Ethics Committee of the Medical Faculty of Eberhard Karls University of Tübingen (Protocol No. 597/2013BO2).

## Author Contributions

NN and DS share the first authorship for this publication. MG and SS did the data collection and -analysis for Site 2 (Hessisch Oldendorf). JS, PB, and FM-D did the data collection and -analysis for Site 1 (Tübingen). JDR coordinated and contributed to the new audiodesign at Site 2. UZ is senior author and PI for Site 1. JR and EA are senior authors and PIs for Site 2.

## Conflict of Interest

The authors declare that the research was conducted in the absence of any commercial or financial relationships that could be construed as a potential conflict of interest. The reviewer TS declared a past co-authorship with one of the authors EA to the handling Editor.

## Footnotes

1 www.xsens.com

2 www.leapmotion.com
