# Supplementary material for: Musical Sonification of Arm Movements in Stroke Rehabilitation Yields Limited Benefits
Source: Front Neurosci. 2019 Dec 20;13:1378. doi: 10.3389/fnins.2019.01378 (PMC6933006; doi:10.3389/fnins.2019.01378)
Supplement: Supplementary file 1 [file Table_1.pdf]

Table S1. *Point estimates (median) of posterior distributions in the tested outcome variables, along with 90 percent credible intervals (quantile-based; Q5 and Q95) for sites 1 and 2, after the intervention.*

| Site | Variable | Intercept | Est. Err | Q5 | Q95 | pre-score | Est. Err | Q5 | Q95 | Group.c | Est. Err | Q5 | Q95 | pre-score:Group.c | Est. Err | Q5 | Q95 |
|---|---|---|---|---|---|---|---|---|---|---|---|---|---|---|---|---|---|
| 1 | ARAT | 14.48 | 3.62 | 8.41 | 20.28 | -4.12 | 2.40 | -8.12 | -0.27 | -0.37 | 1.24 | -2.39 | 1.51 | 0.32 | 1.06 | -1.39 | 2.03 |
| 1 | BBT | 10.08 | 2.92 | 4.78 | 14.26 | -2.10 | 2.17 | -6.27 | 0.60 | -0.50 | 1.30 | -2.77 | 1.36 | 0.10 | 1.09 | -1.63 | 1.88 |
| 1 | invNHPT | 73.95 | 44.65 | -0.25 | 117.27 | -10.57 | 17.40 | -40.54 | 1.50 | -0.09 | 1.38 | -2.19 | 1.92 | -0.03 | 1.48 | -2.33 | 2.31 |
| 1 | TLT | -0.73 | 0.81 | -1.89 | 0.44 | 0.01 | 1.67 | -2.33 | 2.36 | 0.56 | 0.68 | -0.54 | 1.69 | 0.38 | 1.22 | -1.45 | 2.49 |
| 1 | FM.A.D | 0.12 | 0.05 | 0.04 | 0.20 | -0.11 | 0.05 | -0.18 | -0.02 | -0.10 | 0.10 | -0.26 | 0.05 | -0.03 | 0.10 | -0.19 | 0.13 |
| 1 | FM.H | 0.02 | 0.02 | -0.01 | 0.05 | -0.05 | 0.02 | -0.08 | -0.02 | 0.00 | 0.04 | -0.06 | 0.06 | 0.05 | 0.04 | 0.00 | 0.11 |
| 1 | FM.I | -0.02 | 0.13 | -0.24 | 0.10 | 0.04 | 0.30 | -0.23 | 0.55 | 0.03 | 0.26 | -0.21 | 0.48 | -0.08 | 0.60 | -1.11 | 0.47 |
| 1 | FM.J | -0.01 | 0.04 | -0.06 | 0.05 | -0.08 | 0.05 | -0.16 | 0.01 | -0.07 | 0.07 | -0.19 | 0.03 | -0.02 | 0.11 | -0.18 | 0.16 |
| 1 | SIS.1 | 14.72 | 5.09 | 6.42 | 23.07 | -1.63 | 2.95 | -7.90 | 1.20 | -0.06 | 1.53 | -2.44 | 2.14 | -0.24 | 1.61 | -2.75 | 1.95 |
| 1 | SIS.2 | -4.35 | 5.83 | -14.64 | 3.90 | -0.22 | 1.48 | -2.45 | 1.67 | 0.06 | 1.62 | -2.28 | 2.89 | -0.19 | 1.58 | -2.44 | 1.94 |
| 1 | SIS.3 | 7.93 | 3.34 | 2.53 | 13.49 | -0.35 | 1.40 | -2.77 | 1.68 | -0.08 | 1.46 | -2.30 | 2.23 | -0.23 | 1.54 | -2.71 | 1.88 |
| 1 | SIS.4 | -2.00 | 3.60 | -7.81 | 3.94 | -0.84 | 1.56 | -3.57 | 1.22 | 0.41 | 1.71 | -1.70 | 3.11 | -0.43 | 1.65 | -3.10 | 1.67 |
| 1 | SIS.5 | 13.72 | 5.94 | 4.05 | 23.60 | -0.53 | 1.87 | -3.55 | 1.57 | -0.32 | 1.70 | -2.84 | 1.86 | -0.02 | 1.57 | -2.32 | 2.37 |
| 1 | SIS.6 | 21.05 | 4.55 | 13.50 | 28.42 | -3.15 | 4.29 | -11.97 | 1.01 | -0.75 | 2.59 | -4.13 | 1.63 | 0.17 | 1.51 | -1.86 | 2.50 |
| 1 | SIS.7 | 35.61 | 7.22 | 23.86 | 47.40 | -0.01 | 1.55 | -2.34 | 2.25 | -0.18 | 1.60 | -2.56 | 2.04 | 0.04 | 1.56 | -2.21 | 2.42 |
| 1 | SIS.8 | 6.26 | 8.37 | -7.37 | 19.97 | -0.70 | 2.18 | -3.90 | 1.60 | -0.09 | 1.61 | -2.53 | 2.20 | -0.67 | 3.71 | -3.44 | 1.89 |
| 1 | SIS.9 | 12.59 | 7.54 | 0.17 | 24.91 | -0.69 | 2.59 | -3.89 | 1.70 | -0.40 | 2.13 | -3.14 | 2.00 | 0.23 | 1.73 | -2.02 | 2.67 |
| 1 | SIS.total | 111.33 | 28.12 | 65.23 | 157.26 | 0.01 | 1.63 | -2.33 | 2.36 | -0.08 | 1.68 | -2.49 | 2.25 | 0.05 | 1.62 | -2.29 | 2.42 |
| 2 | ARAT | 2.81 | 1.81 | -0.16 | 5.68 | -1.10 | 1.07 | -3.06 | 0.43 | 1.28 | 1.79 | -0.88 | 4.69 | -0.95 | 1.38 | -3.51 | 0.92 |
| 2 | BBT | 4.49 | 2.42 | 0.62 | 8.54 | -0.41 | 1.18 | -2.45 | 1.32 | -0.43 | 1.51 | -3.03 | 1.58 | 0.02 | 1.31 | -2.09 | 1.91 |
| 2 | invNHPT | 8.38 | 7.00 | -4.31 | 18.99 | -0.50 | 1.44 | -2.63 | 1.58 | -0.29 | 1.52 | -2.72 | 1.64 | 1.59 | 5.56 | -1.93 | 9.28 |
| 2 | TLT | 0.50 | 0.26 | 0.08 | 0.92 | -0.07 | 0.21 | -0.41 | 0.27 | 0.52 | 0.45 | -0.21 | 1.24 | -0.04 | 0.37 | -0.65 | 0.56 |
| 2 | FM.A.D | 0.05 | 0.02 | 0.01 | 0.08 | -0.03 | 0.02 | -0.06 | 0.00 | 0.09 | 0.04 | 0.03 | 0.16 | -0.02 | 0.04 | -0.08 | 0.04 |
| 2 | FM.H | 0.00 | 0.03 | -0.05 | 0.05 | -0.08 | 0.05 | -0.15 | -0.01 | 0.06 | 0.06 | -0.04 | 0.17 | -0.01 | 0.09 | -0.16 | 0.14 |
| 2 | FM.I | 0.02 | 0.01 | 0.00 | 0.04 | -0.05 | 0.02 | -0.07 | -0.02 | 0.00 | 0.03 | -0.04 | 0.04 | -0.01 | 0.03 | -0.06 | 0.04 |
| 2 | FM.J | 0.00 | 0.01 | -0.02 | 0.03 | -0.02 | 0.01 | -0.04 | 0.01 | 0.07 | 0.03 | 0.03 | 0.12 | -0.10 | 0.03 | -0.15 | -0.06 |
| 2 | SIS.1 | 6.93 | 2.65 | 2.65 | 11.26 | -11.92 | 2.72 | -16.03 | -7.28 | 0.39 | 1.48 | -1.65 | 2.92 | -0.12 | 1.34 | -2.29 | 1.88 |

| | | | | | | | | | | | | | | | | | |
|---|---|---|---|---|---|---|---|---|---|---|---|---|---|---|---|---|---|
| 2 | SIS.2 | 0.50 | 2.24 | -3.18 | 4.15 | -1.83 | 1.86 | -5.45 | 0.53 | 0.29 | 1.43 | -1.73 | 2.66 | -0.15 | 1.41 | -2.42 | 1.95 |
| 2 | SIS.3 | 4.36 | 2.57 | 0.31 | 8.69 | -0.94 | 1.52 | -3.86 | 1.03 | 0.37 | 1.43 | -1.60 | 2.81 | -0.15 | 1.44 | -2.49 | 1.95 |
| 2 | SIS.4 | 2.29 | 2.34 | -1.51 | 6.02 | -6.04 | 3.46 | -11.79 | -0.50 | 0.34 | 1.44 | -1.77 | 3.06 | 0.56 | 1.49 | -1.38 | 3.14 |
| 2 | SIS.5 | 6.52 | 2.43 | 2.48 | 10.46 | -0.29 | 1.21 | -2.42 | 1.57 | 2.55 | 3.82 | -0.96 | 11.37 | -0.10 | 1.78 | -2.74 | 2.38 |
| 2 | SIS.6 | 2.15 | 3.51 | -3.63 | 7.90 | -1.40 | 2.14 | -5.70 | 1.00 | 0.13 | 1.45 | -2.04 | 2.44 | -0.12 | 1.47 | -2.44 | 2.05 |
| 2 | SIS.7 | 5.62 | 3.75 | -0.50 | 11.80 | -0.57 | 1.48 | -3.18 | 1.44 | -0.06 | 1.38 | -2.27 | 2.06 | -0.10 | 1.46 | -2.41 | 2.08 |
| 2 | SIS.8 | 3.70 | 2.81 | -0.91 | 8.33 | -3.15 | 2.72 | -8.32 | 0.23 | -0.11 | 1.41 | -2.33 | 2.00 | -0.40 | 1.52 | -2.94 | 1.71 |
| 2 | SIS.9 | 13.38 | 3.23 | 8.30 | 18.88 | -5.57 | 3.83 | -12.17 | -0.08 | 0.28 | 1.42 | -1.81 | 2.80 | -0.83 | 1.85 | -3.95 | 1.28 |
| 2 | SIS.total | 57.91 | 13.56 | 35.59 | 80.12 | -0.53 | 2.28 | -3.43 | 1.78 | 0.11 | 1.73 | -2.22 | 2.60 | 0.04 | 1.73 | -2.20 | 2.37 |
