# Supplementary material for: Musical Sonification of Arm Movements in Stroke Rehabilitation Yields Limited Benefits
Source: Front Neurosci. 2019 Dec 20;13:1378. doi: 10.3389/fnins.2019.01378 (PMC6933006; doi:10.3389/fnins.2019.01378)
Supplement: Supplementary file 2 [file Table_2.pdf]

Table S2. *Predictive accuracy. Models are listed in decreasing order of predictive accuracy, as assessed by Pareto-smoothed leave one out cross-validation, and expressed in differences in expected posterior log-likelihood. SE, standard error.*

Model #	Difference	SE
5	0.0	0.0
4	-0.7	1.2
6	-0.7	0.2
7	-1.0	0.3
1	-3.8	3.9
2	-6.7	3.9
3	-7.7	3.8
